# Practitioners’ perceptions on ecological restoration: activities, challenges and solutions on two small oceanic islands

**DOI:** 10.7717/peerj.21687

**Published:** 2026-09-21

**Authors:** François M.M.P. Baguette, Cláudia Baider, Raphael Merven, François Benjamin Vincent Florens

**Affiliations:** 1 Tropical Island Biodiversity, Ecology and Conservation Pole of Research, Department of Biosciences and Ocean Studies, Faculty of Science, University of Mauritius, Le Réduit, Mauritius; 2 The Mauritius Herbarium, Agricultural Services, Ministry of Agro-Industry, Food Security, Blue Economy and Fisheries, Le Réduit, Mauritius

**Keywords:** Biodiversity conservation, Conservation management, Human dimension, Mauritius, Restoration ecology, Restoration effectiveness, Restoration monitoring, Restoration success, Rodrigues, Social survey

## Abstract

Ecosystem degradation worldwide causes biodiversity loss and negatively impacts their functioning, eroding the services they provide to the detriment of society and human well-being. The magnitude of this crisis makes ecological restoration a global priority, but progress has been hampered by a variety of factors including a low uptake of evidence-based management by practitioners, limited monitoring of restoration success, and social and political drivers occurring in competitive, multi-stakeholder management contexts. Exploring practitioners’ opinions, perceptions and motivations can thus provide useful insights and identify barriers to upscale ecological restoration and increase its effectiveness. This study aimed to assess the rationale behind the implementation of current restoration activities, and characterise the barriers to adopt improved approaches that have been shown to be more cost-effective. Using a questionnaire-based survey on Mauritius and Rodrigues oceanic islands, both well-known in the fields of terrestrial ecological restoration and conservation, we explored (1) the modalities of restoration activities implemented for native terrestrial habitats conservation, (2) challenges impacting their implementation, and (3) potential solutions from conservation practitioners’ perspectives. We interviewed 54 conservation practitioners, conducted a hierarchical clustering analysis to categorise the restoration activities they implement, and identified the most frequently perceived challenges hindering them, along with the perceived solutions foreseen to improve restoration success. Four clusters of activities were identified. Restoration activities are dominated by the control of invasive species, *in-situ* planting of native plants propagated in nurseries, and ecological monitoring activities, while research, fauna conservation, and policy enforcement are implemented by a minority of practitioners. Conservation practitioners describe many challenges that hinder their activities, most of which arising at the organisation and national (including governance) levels. Most solutions perceived to improve restoration success, however, are technical and site-specific (*e.g.*, use of specific equipment, better methods to control invasive species, new technology). It is therefore necessary to better structure the efforts implemented to adequately address predominant challenges, and clarify what indicators are used to measure restoration success to ensure a robust evaluation of restoration projects. This assessment of conservation practitioners’ perceptions and actions can be used as a tool to leverage improvements in restoration activities for biodiversity conservation, and shift the current ecological restoration approaches towards more cost-effective and research inclusive solutions that will enable the upscaling of ecological restoration that is required if sustainable conservation is to be achieved.

## Introduction

Globally, human-driven biodiversity loss is among the most critical environmental crises currently faced by humanity (Baillie, Hilton-Taylor & Stuart, 2004; Vie, Hilton-Taylor & Stuart, 2009; Barnosky et al., 2011), resulting in ecosystems being degraded (Worm et al., 2006; Wardle et al., 2011), along with the services they provide (Pimm et al., 2014; Cardinale et al., 2012; Costanza et al., 2014). Wildlife populations have declined by ∼73% since 1975 (WWF, 2024), with one million species being threatened with extinction (IPBES, 2019), and ecosystem collapse could cost ∼2.3% of annual global GDP by 2030 (Johnson et al., 2021). The magnitude of the crisis made ecological restoration a global priority (Aronson & Alexander, 2013; Jørgensen, 2015). The Aichi Targets, established in 2010 by the Convention on Biological Diversity Secretariat, called all member states to restore 15% of degraded or deforested areas by 2020, emphasising recovery of biodiversity (CBD, 2011). In 2011, the Bonn Challenge was launched to initially restore 150 million hectares of degraded forest by 2020, updated to 350 million hectares by 2030 (Pistorius & Freiberg, 2014; UN Climate Summit, 2014). However, upscaling restoration to align with stated aims of countries remains challenging (Florens, 2013; Simkins et al., 2025), resulting in global targets shortfalls (Hoffmann et al., 2010; Pimm et al., 2014; Tittensor et al., 2014; Waring, 2024). There is thus an urgent need to better understand the constraints and challenges besetting effective ecological restoration worldwide (Choi et al., 2008; Suding, 2011; Perring et al., 2015; Murcia et al., 2016).

The limited use of science-based approaches within ecological restoration projects (Van Diggelen, Grootjans & Harris, 2001; Halle, 2007; Walsh, Dicks & Sutherland, 2015; Nature, 2026) has been identified as one of those challenges, often slowing achievements of national and international policy targets (Aronson & Alexander, 2013; Murcia et al., 2016; Perring, Erickson & Brancalion, 2018). In practice, this translates into limited uptake of international guidelines for the implementation and monitoring of restoration activities. Such guidelines include those of the Society for Ecological Restoration International Primer on Ecological Restoration (SER, 2004), or the International Principles and Standards for the Practice of Ecological Restoration (Gann et al., 2019). This situation has been partly attributed to a perceived disconnect between conservation research, policy and practice (Arlettaz et al., 2010; Baker & Eckerberg, 2013; Aguilar-Garavito et al., 2025), and the view that on-the-ground practical constraints may be underestimated (Knight et al., 2008; Hulme, 2014; Ottewell et al., 2016), underscoring the importance of incorporating human perceptions and locally grounded perspectives in decision-making (Sherring, 2023).

The perspectives of practitioners are important for achieving ecological objectives and minimise potential disagreements (Elias et al., 2022; Mansourian et al., 2025a). Indeed, scientific and technical discourses often overlook the social processes of ecological restoration (Löfqvist et al., 2023), resulting in a partly blinded vision of restoration equity, effectiveness and multidimensional impacts’ processes that shape its outcomes (Elias et al., 2022; Mansourian et al., 2025a). For example, the way practitioners define ecological restoration might vary from a purely ecological and functional definition (Hall, 2005; Young, Petersen & Clary, 2005; Jordan & Lubick, 2011) to one considering social aspects (Higgs, 1994; Hobbs et al., 2004), modulating the chosen restoration activities (Martin, 2017). Furthermore, humans have different ideals, intuitions, moral considerations, institutions and hold values shaped by diverse cosmovisions or worldviews, perspectives, and knowledge systems (Pascual et al., 2023; Wienhues, Luuppala & Deplazes-Zemp, 2023). Practitioners’ perception of ecological restoration therefore varies with the degradation state of the ecosystems they manage (Meli et al., 2017), highlighting a reciprocal process embedded in complex and evolutive feedback loops, typical of socio-ecological systems (Ostrom, 2009; Li, Dong & Liu, 2020). These differences lead to diverse valuations of biodiversity (Weikard, 2002; Salles, 2011; Verma, 2016) resulting in the implementation of different practices with a variable perception of effectiveness (Latombe et al., 2022; Löfqvist et al., 2023). Hence, it is crucial to adopt a multidimensional approach to understand the drivers of current restoration activities, provide effective recommendations, and improve the evaluation of ecological restoration success.

Here, we focussed on oceanic islands, where biodiversity loss is exacerbated (Caujapé-Castells et al., 2010; Spatz et al., 2017; Howard, Flather & Stephens, 2020; Leclerc et al., 2020), and chose Mauritius and Rodrigues, two islands much impacted by human-activities (Gade, 1985; Florens, 2013; Hammond et al., 2015). Since the 1980s, most of the restoration work implemented there has favoured intensive and expensive restoration methods (Mauremootoo, 2003; Jones, 2004; Sodhi et al., 2011). Such an approach was often justified (*e.g.*, Impey, Côté & Jones, 2002; Hugel, 2012), but in parallel, the need for more cost-effective solutions to up-scale restoration projects has long been recognised yet somewhat resisted (Florens & Baider, 2013). Upscaling restoration is vital because the conservation successes so far are still overshadowed by a much greater concurrent loss of biodiversity (Baider et al., 2010; Bissessur, Baider & Florens, 2017; Florens et al., 2017), which can be largely addressed if cheaper and better restoration approaches are used instead (Florens et al., 2010; Baider & Florens, 2011; Baguette, Baider & Florens, 2025a; Baguette, Baider & Florens, 2025b). Nevertheless, propagation and planting of native species for example (and the perception of the need of such technique) is still preferred among practitioners despite carrying several weaknesses (Florens & Baider, 2013). Likewise, cost-effective solutions are rarely applied (Florens & Baider, 2013), highlighting the need to understand why and identify the barriers to the uptake of alternative methodologies.

We adopted a structured questionnaire-based survey with conservation practitioners from different sectors (government institutions, non-governmental organizations (NGOs), private sector, individuals, academia) using Mauritius and Rodrigues islands as case studies. The aim was to assess the rationale behind the implementation of the current restoration activities in Mauritius and Rodrigues, and characterise the barriers to adopt improved approaches that have been shown to be more cost-effective. To do that, we assessed and characterised (1) the modalities of activities implemented to restore native terrestrial habitats; (2) the challenges conservation practitioners face; and (3) the potential solutions that could be brought based on practitioner’s perceptions. In this case, we consider that ecological restoration for biodiversity conservation inherently links habitat recovery with species protection. Indeed, conservation interventions facilitate ecosystem recovery, while restored habitats enhance biodiversity persistence and resilience (Benayas et al., 2009; Gann et al., 2019). Hence, a better understanding of restoration viewed from the perspective of conservation practitioners can offer insights to advance the effectiveness of restoration (understood as how effectively activities implemented contribute to meet set objectives) and help identify research gaps (Mansourian & Vallauri, 2014). Those could therefore increase the effectiveness of the conservation dollar in sustainably conserving threatened biodiversity, improving the alignment of restoration with broader goals and strategies (Gavin et al., 2018; Guariguata & Evans, 2020; White et al., 2023). Ultimately, this would facilitate the upscaling of restoration activities, thereby increasing opportunities for broader implementation.

## Materials and Methods

### Study site

**Figure 1:**
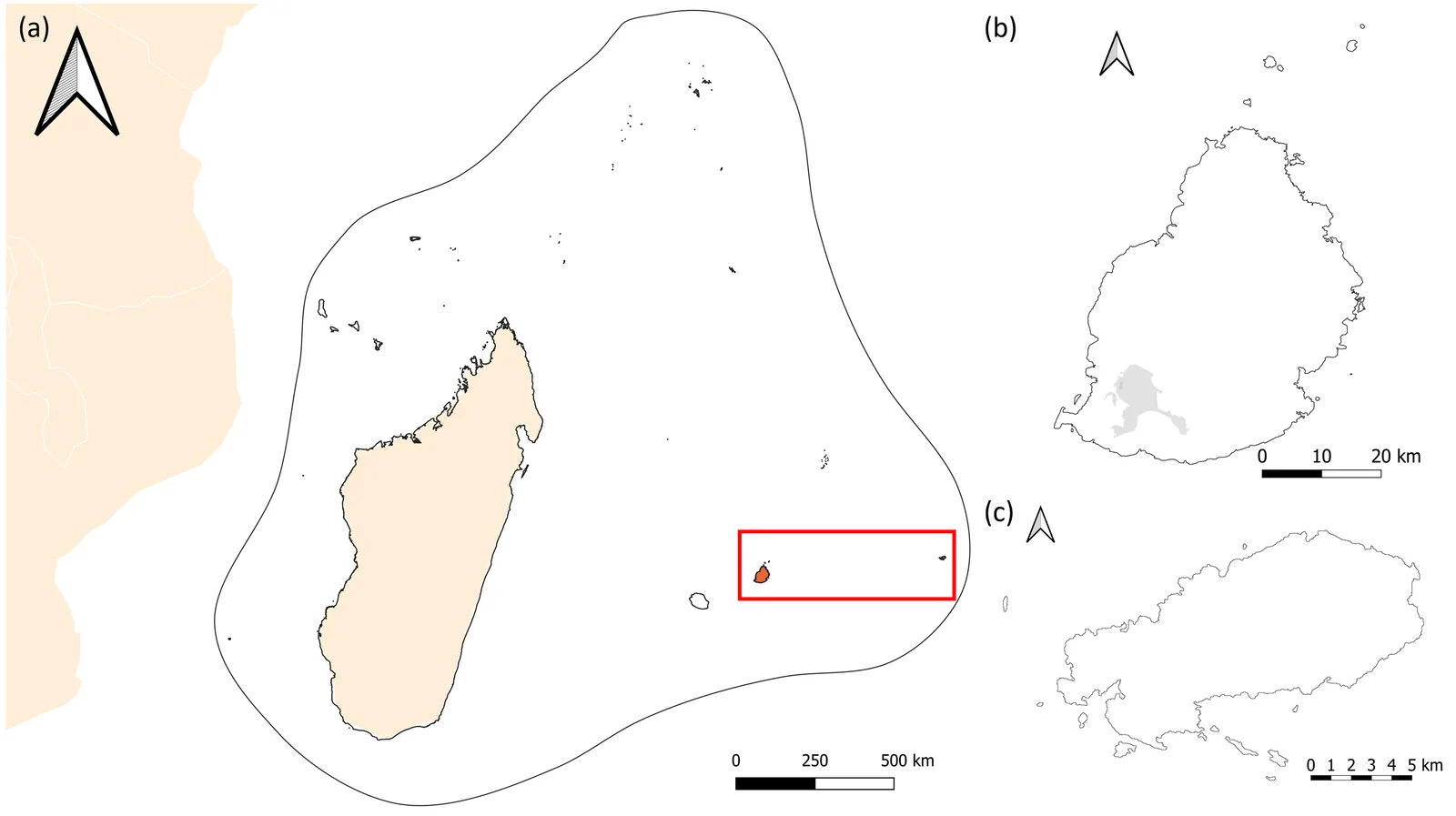
The study sites of Mauritius and Rodrigues, displayed at different geographical scales. (A) Biodiversity hotspot of Madagascar and the Indian Ocean Islands (circled), with the location of Mauritius and Rodrigues Islands in red rectangle. (B) Mauritius Island with the Black River National Park shaded. (C) Rodrigues Island. Scale bars in kilometers.

Mauritius (1,865 km^2^) and Rodrigues (108 km^2^) are two volcanic oceanic islands from the Mascarene archipelago located within one of the world’s biodiversity hotspots (Myers et al., 2000) (Fig. 1). Since human’s first colonisation in the 17th century, both islands were transformed into some of the most human-impacted places on Earth, with virtually no native terrestrial habitat left on Rodrigues (Gade, 1985) and only 4.4% of the original habitats surviving in fragmented patches on Mauritius (Hammond et al., 2015). The dire situation that this created for their native biota triggered growing conservation management and ecological restoration attention mainly since the 1970’s (Cheke & Hume, 2008) which in turn generated internationally useful conservation science (Temple, 1974; North & Bullock, 1986; Jones et al., 1995; Cheke & Hume, 2008). In more recent decades, Mauritius and Rodrigues continued contributing to conservation science worldwide, like in pioneering the use of analogue species to replace lost ecological function (Griffiths et al., 2010), exploring the consequences of extinctions (Albert et al., 2021; Heinen et al., 2023), and implementing native species reintroductions (Impey, Côté & Jones, 2002; Davies et al., 2018). The Government of Mauritius supported ecological restoration and biodiversity conservation efforts as signatory of various international conventions, and by enacting environmental protection laws (Government of Mauritius, 2021). Nevertheless, Mauritius’ natural capital continues to decline (Florens, 2013; Koenig & Deenapanray, 2024), even within protected areas (Florens et al., 2017; Bissessur et al., 2023), despite significant financial support from international donors.

### Data collection

The study was conducted and reported in accordance with the “Strengthening the Reporting of Observational Studies in Epidemiology” (STROBE) guidelines (Von Elm et al., 2007 see Supplemental Information 1 for completed checklist). A structured questionnaire-based survey was applied to conservation practitioners from Mauritius and Rodrigues involved in ecological restoration of native terrestrial habitats (including wetlands). For eligibility, practitioners had to be legal individuals (≥18 years old) who were or are active in the field of terrestrial ecological restoration within at least one of the two islands. The questionnaire consisted of both closed and open-ended questions, alongside Likert-scales statements and free-listing questions (Burt et al., 2022; Wienhues, Luuppala & Deplazes-Zemp, 2023). It was structured in six parts: part 1 focused on practitioners’ background information and organisation; part 2 on practitioners’ perceptions on nature; part 3 on implemented restoration activities and their modalities; part 4 on challenges and constraints faced by practitioners to implement on-ground restoration activities; and parts 5 and 6 at identifying potential solutions to improve restoration activities and answer mentioned challenges (English version in Supplemental Information 2).

The questionnaire was piloted with 12 conservation practitioners from other islands of the Western Indian Ocean between July and August 2024. Based on the responses and feedback, the questionnaire was revised, and the final study methodology received approval by the University of Mauritius Research Ethics Committee in September 2024 (Ref No: UoMREC/2024/P76). Subsequently, a list of representatives of all key stakeholders involved in ecological restoration in Mauritius and Rodrigues was drawn by the authors, based on their expertise, to identify potential candidates. Invitations were then sent by email or letter. Prior to survey implementation, respondents were asked to give their written and oral informed consent to the study using a Consent Form (Supplemental Information 3). Specific precautions were taken regarding the study purposes and risk disclosure, anonymity and confidentiality measures, the conditions of respondents’ participation and data protection. From October 2024 to May 2025, 89 practitioners were invited and 54 persons accepted and were interviewed (60.6% positive response rate). This represents a sufficient and representative sample size following purposive sampling (Martínez-Mesa et al., 2016; Van Rijnsoever, 2017; Vasileiou et al., 2018). Respondents were individually interviewed in person, or interviewed remotely when necessary. On average, each survey lasted ∼90 min, and was recorded in writing and using a handheld audio recorder. The survey was run in either English or French, as per the participant’s preference, to maximise participation. At the end of each interview, a snowball sampling approach was implemented to seek new participants based on respondents’ network and knowledge (Parker, Scott & Geddes, 2019).

### Data analysis

Raw data were computed in English (the first author translated French answers) in Microsoft Excel spreadsheets. For this study, data from part 1 of the survey were summarized to compile descriptive information about the study participants, their role(s), and the organisation(s) they work(ed) for. Responses from questions 20, 21, 23, and 53 of the questionnaires were used to summarize the diversity of restoration activities implemented. Responses to the open-ended questions 20, were qualitatively coded and categorized into distinct thematic variables. Each identified theme was entered as a separate binary (presence/absence) or categorical variable in the dataset, allowing for quantitative analysis of the qualitative data. In addition, responses to closed-ended (yes/no) questions 21, 23, and 53 were included as binary variables, resulting in a final dataset comprising 32 variables (Supplemental Information 4).

A Multiple Correspondence Analysis (MCA) was done using 17 of the 32 binomial or categorical variables extracted to categorize the diversity of restoration activities implemented (Supplemental Information 5) after variables cited fewer than five times were excluded. The final selection was informed by a Cramer’s V correlation analysis. This method allowed the identification of fewer independent latent variables, constructed as linear combinations of the original variables, that represent underlying concepts not directly measurable in the original dataset. The resultant dimensions (or components) obtained from the MCA (for each practitioner) were then utilized in the Hierarchical Clustering Analysis (HCA), using Ward’s minimum variance method (Ward Jr, 1963), allowing homogenous clusters of practitioners to be identified. Ward’s linkage method groups observations into a cluster to reduce within-cluster variation, ensuring the least increase in the error sum of squares.

To summarise the challenges faced by conservation practitioners, responses from the open questions 32, 35a, 38a, 40b, 41b, 43a, and 45a were grouped based on topic (*e.g.*, resource constraints or limited staff capacity) and broader content themes (*e.g.*, national research strategy or collaboration & communication). The frequency of each topic and theme that was mentioned was tallied. If several responses falling within the same topic were mentioned within one question, the topic was recorded as mentioned once. However, if responses falling within the same topic were mentioned under different thematic questions (*e.g.*, affecting practitioner ability to develop collaborations AND to reach a sufficient staff capacity), the number of thematic questions it appeared in was recorded as number of mentions. To summarise the solutions foreseen by conservation practitioners to improve restoration success (understood as the successful completion of the restoration of a site or a restoration project), responses from the open questions 46a and 47 were grouped similarly based on topic (*e.g.*, develop more collaboration or train staff) and broader content themes (*e.g.*, technical solution or project development), and the same calculation of frequencies was applied.

Challenges and solutions were further categorized according to the level at which they emerged and were implemented respectively, specifying levels as national, organizational, or site/project level. For example, when a practitioner referred to “low government efficiency”, this challenge was coded as occurring at the national level, because biodiversity protection falls under the country’s national jurisdiction. Conversely, if a practitioner described a “lack of water access” this was coded as a challenge arising at the individual site/project level. Each topic was thus logically classified into one of three levels: (a) national (*e.g.*, policy and legislation), (b) organisational (*e.g.*, staffing and resources), or (c) project/site (*e.g.*, site-specific conditions) (see Supplemental Information 6 and 7). In addition, qualitative responses to selected questionnaire items were transcribed and presented verbatim to complement quantitative findings and enrich the discussion.

## Results

A total of 54 conservation practitioners completed the survey, including 61% of male and 39% of female respondents. Conservation practitioners were mostly from non-governmental organisations (NGOs) (*N* = 20, 37%), followed by private companies and subsidiaries (*N* = 14, 25.9%), governmental institutions (*N* = 11, 20.4%), and academia (each *N* = 5, 9.3%). In addition, four practitioners (7.4%) were individuals conducting ecological restoration on their own initiative. Practitioners were mostly involved directly in the implementation of restoration projects (*N* = 52, 96.3%) across a wide range of ecosystems (Supplemental Information 8), while some were only or also acting as advisor or researcher (*N* = 25, 46.3%), and/or involved in policy regulation (*N* = 10, 18.5%), and/or funder or facilitator of ecological restoration projects (*N* = 8, 14.8%), and/or reviewer of restoration project implementations (*N* = 1, 1.9%). Ecological restoration activities applied in terrestrial habitats of Mauritius and Rodrigues were analysed using Multiple Correspondence Analysis (MCA), with the first two dimensions accounting for 29.62% of the total inertia. In all, 13 variables were significantly contributing to the clustering of the activities (significant *p*-values < 0.05) (Supplemental Information 9).

**Figure 2:**
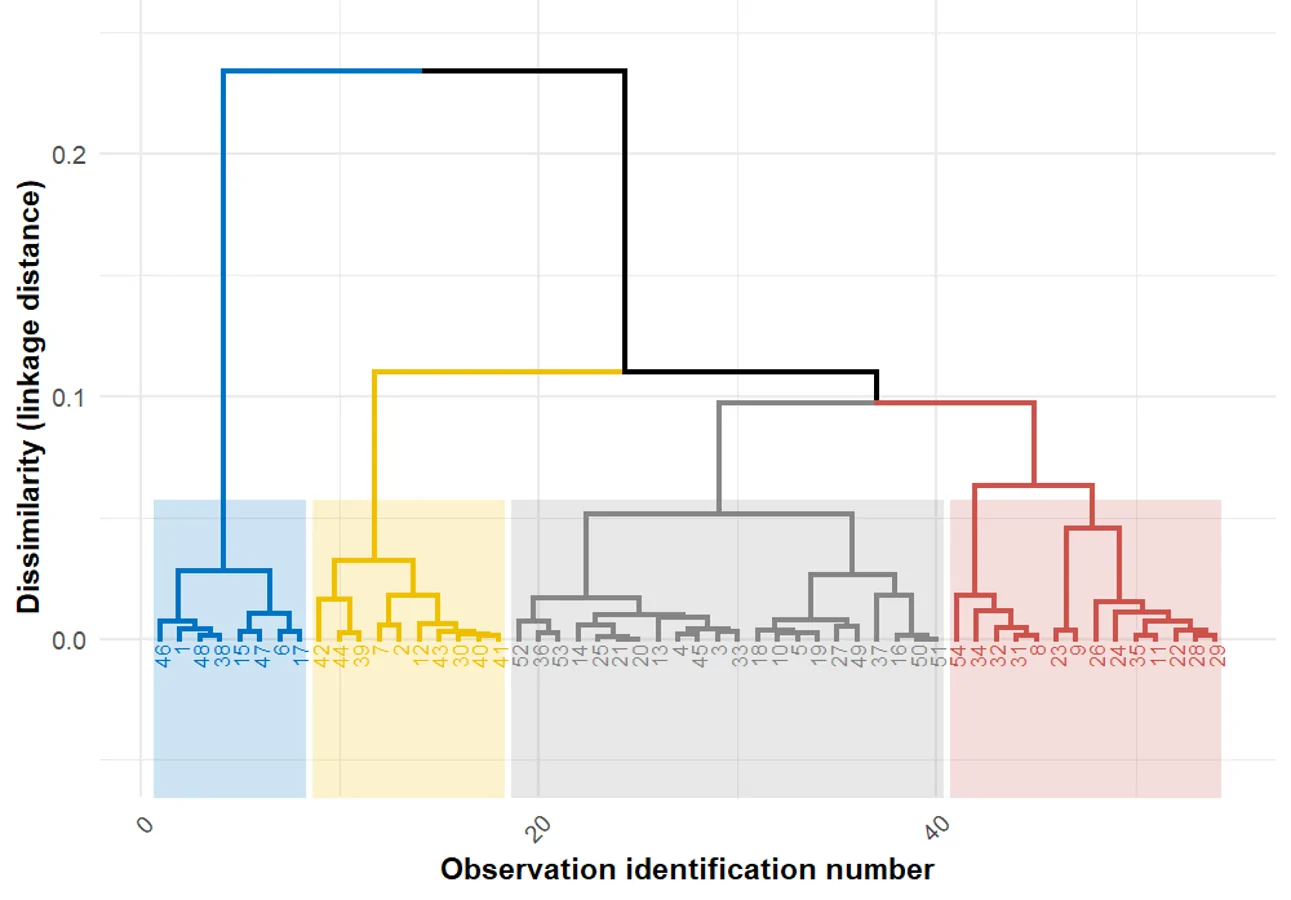
Hierarchical Clustering Analysis plot illustrating the four clusters of ecological restoration practices implemented in Mauritius and Rodrigues. “Cluster 1: fauna-driven” is represented in yellow, “cluster 2: habitat restoration” in grey, “cluster 3: habitat rehabilitation“ in red, and “cluster 4: research” in blue. This plot has been computed based on all restoration activities reported by 54 responding conservation practitioners (see Supplemental Information 10).

We conducted a Hierarchical Clustering Analysis (HCA) using data from the first eight MCA dimensions (representing 70% of explained cumulative variance) to characterise restoration activities and identify clusters (Fig. 2). Four clusters were identified, and each was assigned a name reflecting its main characteristics following its most important variable categories (Table 1). The first one (“fauna-driven”) regroups fauna-focused restoration activities involving predator control activities, fauna conservation programs, and fencing, in addition to weeding and planting activities. Cluster 2 (“habitat restoration”) is centered mostly on planting, propagation, and ecological monitoring, but not including plant after-care like watering, neither fauna conservation programs nor predator control. Cluster 3 (“habitat rehabilitation”) is a cluster with restoration activities focusing on weeding, planting of native and exotic species, and investing in plant after-care like watering, excluding plant propagation. This cluster is also characterised by an absence of utilisation of pioneer plant species as a strategy, predator control, research, and monitoring of socio-economic parameters. Finally, cluster 4 (“research”) is characterised by the use of research as core activity, and the absence of weeding of invasive species, plant propagation, or planting of native vegetation as restoration activities. Of the 17 variables retained for analysis, planting, weeding, monitoring of ecological parameters, and plant propagation were the most frequently implemented, cited by 85.2%, 72.2%, 64.8% and 63% of respondents, respectively. The implementation of research, fauna conservation programs, policy enforcement, and advising on restoration methods were the least common ones, cited by 18.5%, 14.8%, 7.4% and 7.4% of respondents, respectively.

**Table 1:** Main characteristics of the four clusters of restoration practices implemented in Mauritius and Rodrigues identified through hierarchical clustering. ‘% Respondents’ shows the proportion of respondents endorsing each characteristic and ‘*p*-value’ the associated *p*-value of each characteristic.

	Main characteristic	% Respondents	*p*-value
Cluster 1 (“fauna-driven”)	Weeding of invasive species	100%	1.14E−02
	Plant native species	100%	4.47E−02
	Conduct predator control	91.70%	1.48E−09
	Use fencing	91.70%	6.50E−03
	Plant at variable densities	75%	2.09E−02
	Fauna conservation focus	66.70%	4.76E−07
	Develop and implement policies	25%	3.23E−02
Cluster 2 (“habitat restoration”)	Plant native species	100%	2.99E−03
	No fauna conservation focus	100%	1.75E−02
	Propagation of natives in nurseries	95%	1.24E−04
	No predator control	95%	1.99E−02
	No plant after-care	95%	3.34E−02
	Conduct ecological monitoring	85%	1.90E−02
	Plant at low densities	45%	3.16E−05
	Use of pioneer species –unknown	30%	1.50E−03
Cluster 3 (“habitat rehabilitation”)	Do not conduct research	100%	3.54E−02
	No predator control	100%	1.63E−02
	Do not use pioneer species strategically	92.90%	9.75E−04
	Do not conduct socio-economic monitoring	92.90%	3.27E−02
	Do not propagate species in nurseries	71.40%	3.30E−03
	Ensure plant after-care	64.30%	1.72E−05
	Use visual observation data for monitoring	42.90%	1.45E−02
	Include exotic species	35.70%	6.33E−04
Cluster 4 (“research”)	Do not plant	100%	9.61E−10
	Planting density not applicable	100%	4.32E−08
	Do not use fencing	100%	4.71E−04
	Do not conduct weeding of IAS	87.50%	2.54E−04
	Plant species selection not applicable	75%	1.08E−06
	Conduct research	75%	2.01E−04
	Do not propagate species in nurseries	75%	2.62E−02
	Provide advice on restoration methods	37.50%	8.59E−03

### Ecosystem threats mobilising practitioners’ resources

Out of all perceived threats cited to affect the ecosystems managed by conservation practitioners for restoration, 13 were classified as severe, the most cited being: invasive alien species (IAS) (*N* = 45, 40.2%), development (*N* = 16, 14.3%), community related issues (including for example negative perception towards restoration, lack of knowledge and understanding, vandalism and illegal activities) (*N* = 10, 8%), climate change impacts (including for example increased frequency of extreme weather events like droughts and cyclones, sea-level rise) (*N* = 9, 8%), harsh weather conditions (including drought, cyclones, heavy rains) (*N* = 7, 6.3%), habitat degradation and consequences (including heavy habitat destruction, soil erosion, species loss) and pollutions (*N* = 6, 5.4%, respectively). Out of those mobilising most practitioners’ resources (*e.g.*, human and financial), IAS represented the most cited as well (*N* = 36, 48%), followed by community-related issues (*N* = 15, 20%), pollution (*N* = 5, 6.5%), climate change and harsh weather conditions (*N* = 4, 5.3%, respectively).

### Challenges to ecological restoration

**Figure 3:**
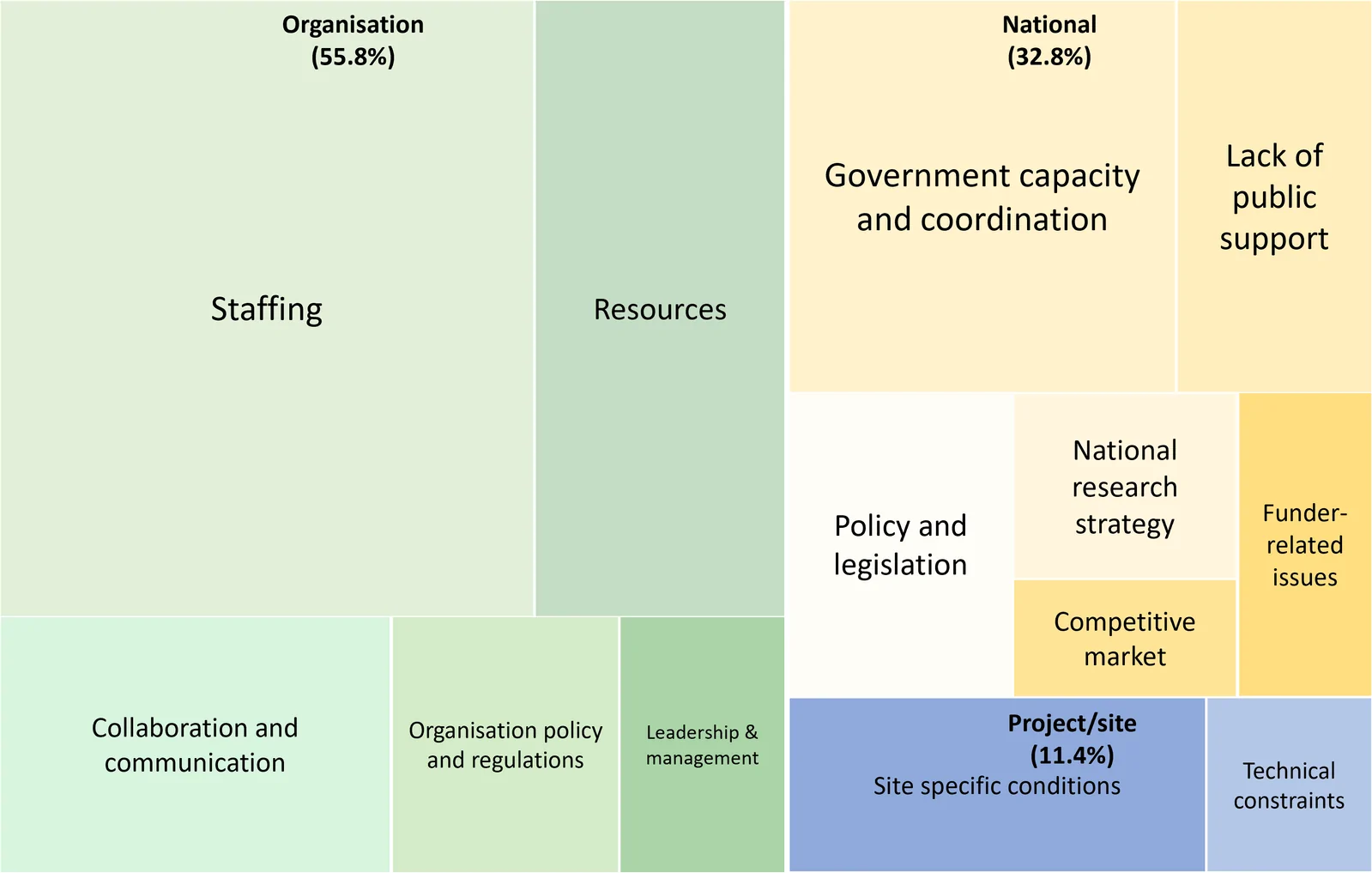
Treemap plot presenting the proportion of challenges for each of the main topics defined, organized by the provenance level associated with each of the challenges. The proportion of challenges that fall into each level is shown below each level. Each block size is proportionate to the number of barriers that topic represents.

**Figure 4:**
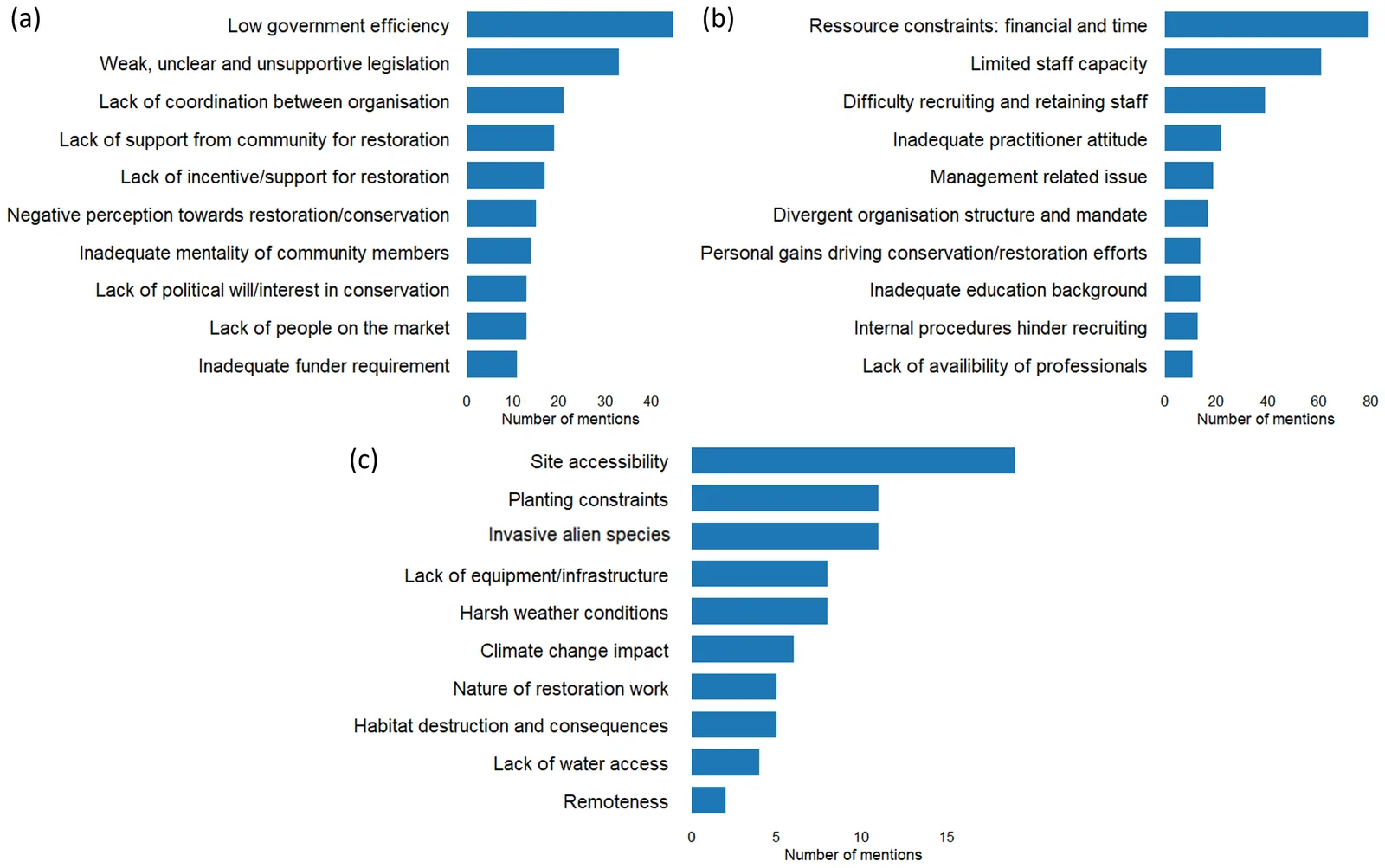
Challenges faced by conservation practitioners in Mauritius and Rodrigues retrieved from 54 interviews. (A) Frequency of the ten most-mentioned challenges arising at the national level. (B) Frequency of the ten most-mentioned challenges arising at the organisation level. (C) Frequency of the ten most-mentioned challenges arising at the project/site level.

We identified 62 distinct challenges under 15 themes raised by conservation practitioners’ as affecting the implementation of their restoration activities by hindering their capacity to attain their restoration objectives, develop collaborations, and ensure sufficient staff capacity (Supplemental Information 6). Among those, the majority of challenges described arose at organisation level (55.8%), followed by those arising at national level (32.8%) and project/site level (11.4%) (Fig. 3). The themes under which practitioners cited most challenges were “Staffing” (*N* = 151), “Government capacity and coordination” (*N* = 80), and “Resources” (*N* = 77) (Fig. 4). At national level, low government efficiency, weak, unclear and unsupportive legislation, and lack of coordination between conservation organisations were the most frequently cited challenges. At organisation level, resource constraints (time and financial), limited staff capacity, and staff recruitment and retention issues were the most cited challenges. At project/site level, site accessibility, planting constraints and IAS were the most cited ones (Fig. 4).

### Improving ecological restoration success

**Figure 5:**
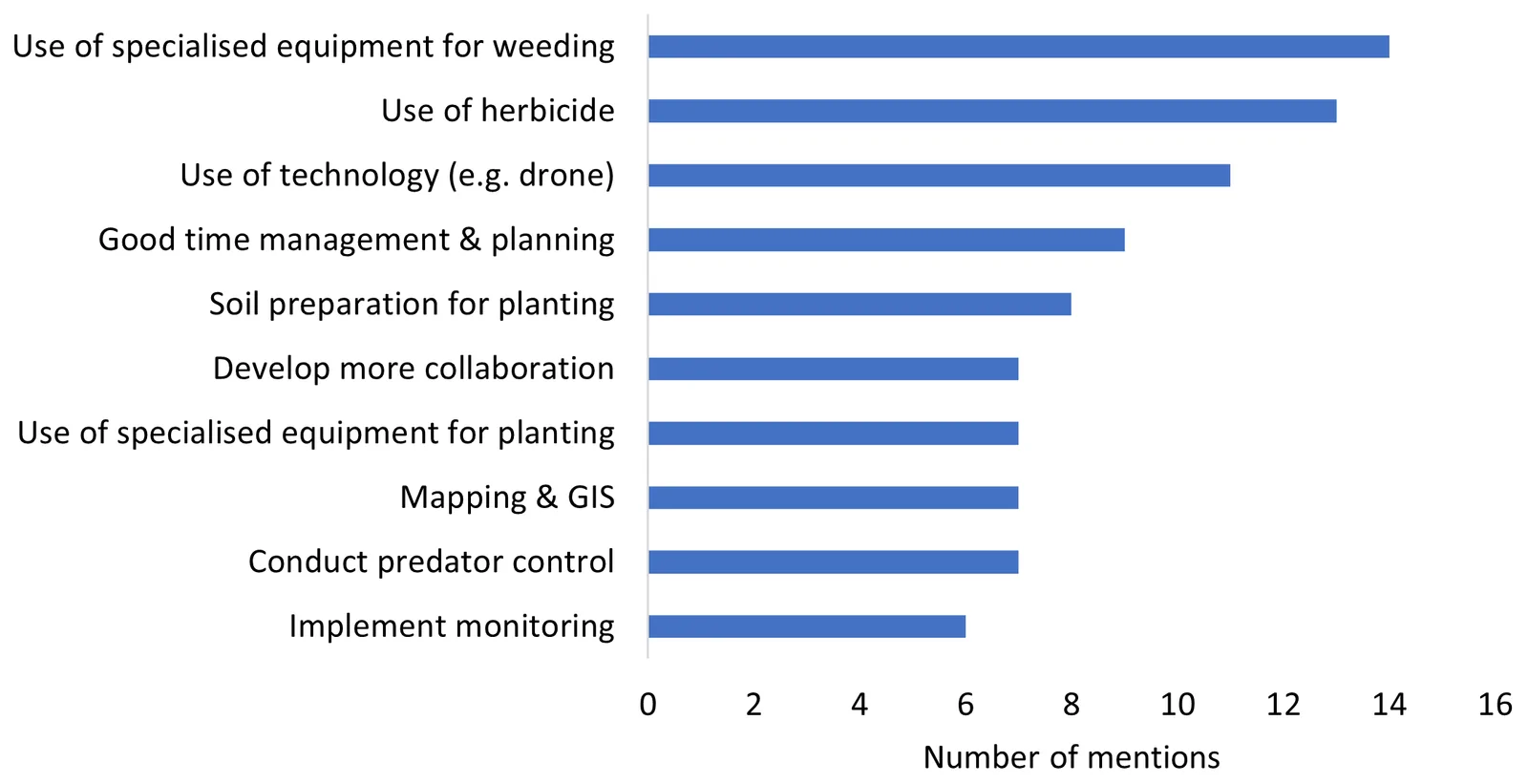
Ten most-mentioned solutions perceived to improve restoration success in the islands of Mauritius and Rodrigues based on 54 interviews with conservation practitioners.

Among all perceived solutions reported to improve restoration success (*N* = 199), the majority are implemented at the project or site level (*N* = 135), followed by those implemented at the organization level (*N* = 45) and national level (*N* = 19). More than two-thirds of the perceived solutions are technical (such as using specific tools for weeding and planting, technology, and herbicides) and methodological (such as circle weeding, uprooting invasive plants, or using pioneer species) (*N* = 135). Besides these, eight additional solution categories were reported, with solutions aiming at improving restoration project management and planning being the most cited (*N* = 15), while project evaluation and monitoring solutions were the least cited (*N* = 2) (Supplemental Information 4). The most cited solutions include the use of specialised equipment for weeding invasive alien plants (*e.g.*, chainsaw, uprooter) (*N* = 14), herbicides (*N* = 13), and technology (*e.g.*, drone and camera traps) (*N* = 11) (Fig. 5).

## Discussion

### Situating restoration activities in Mauritius and Rodrigues within a global context

In our study, we show that restoration activities implemented for biodiversity conservation in Mauritius and Rodrigues can be grouped into four clusters, primarily differentiated by the extent and diversity of activities undertaken. Overall, restoration efforts largely focus on restoring plant communities and habitat structure, which were the core activities in three out of the four clusters identified, rather than associated fauna communities, only represented in the first cluster, a pattern also observed globally (Crouzeilles et al., 2016). Furthermore, projects predominantly rely on active restoration approaches, while only a limited number of practitioners employ more passive strategies such as assisted natural regeneration, reflecting trends documented in other tropical regions (Chazdon, 2008; Holl & Aide, 2011; Meli et al., 2017). Likewise, research, which constituted the defining activity of “cluster 4”, was perceived as an integral component of restoration by fewer than 20% of respondents, consistent with previous studies showing that restoration projects often proceed without strong scientific integration (Hobbs, 2007; Palmer, Zedler & Falk, 2016; Holl & Brancalion, 2020; Nature, 2026). Overall, the landscape of restoration activities in Mauritius and Rodrigues therefore appears broadly consistent with global patterns, underscoring the relevance of these islands as valuable case studies for further investigating the effectiveness of restoration efforts.

Similarly, challenges identified in our study align with the constraints faced by practitioners globally, such as limited funding, governance constraints, insufficient technical knowledge, and limited local communities’ involvement, all of which hinder the scaling up of restoration initiatives (Holl, 2017; Chazdon & Brancalion, 2019; Budiharta & Holl, 2025). Addressing these challenges requires improved stakeholder collaboration, stronger monitoring frameworks, and the adoption of cost-effective restoration strategies such as assisted natural regeneration (Benayas et al., 2009; Moyo et al., 2021; Nance, Clarke & Cook, 2024). In Mauritius and Rodrigues, however, our results show that practitioners do not perceive these approaches as the primary means of improving restoration success. Instead, they emphasise more on technical solutions aimed at enhancing the effectiveness of specific interventions, such as planting or weeding. These perceptions appear symptomatic of restoration efforts implemented in relative isolation by different organisations, each seeking to optimise particular interventions rather than addressing ecological restoration as an integrated process.

This situation, however, raises questions on the overall success of restoration activities implemented in Mauritius and Rodrigues, and how conservation practitioners measure restoration success at site and national level. During interviews, we reported very different definitions of restoration success between conservation practitioners, some of them not being able to provide any clear definition of success, others defining it simply as “the restoration of a degraded area into beautiful and peaceful green spaces”, and others adopting more science-based definitions emphasising on concepts such as restored areas functionality, increased resilience, restored natural regeneration, and cost-effectiveness of activities. These observations suggest diverse interpretation and measures of restoration success ranging from subjective to objective and evidence-based, and driven by other factors that may not necessarily align with what is best for biodiversity conservation. They also highlight the need to more systematically consider human dimensions in ecological restoration research and practice (Egan, Hjerpe & Abrams, 2012; Mansourian et al., 2024; Mansourian et al., 2025a; Mansourian et al., 2025b)

### Implications of current restoration activities & perceptions for biodiversity conservation

Conservation practitioners in Mauritius and Rodrigues use multiple and different approaches to restore native terrestrial ecosystems. Most interviewed practitioners favour intensive interventions like native species propagation and planting, and only few prioritise assisted natural regeneration methods like the use of pioneer plants following weeding activities. Additionally, although most practitioners (*N* = 44; 81%) propagate only native plants, some (*N* = 5; 9%) reported using unspecified exotic species to mitigate soil erosion, and for their aesthetic, medicinal, or food value. This variety of approaches may stem from ecological restoration being undertaken by diverse stakeholders with varied backgrounds, degrees of expertise, motivations, moral foundations and values (IPBES, 2022) and to some extent, objectives. This situation makes the definition of success somewhat problematic, which in turn generates barriers in achieving and reporting national targets and priorities. This plurality of restoration approaches and clusters highlights how the field operates within complex political and technical arenas (Wienhues, Luuppala & Deplazes-Zemp, 2023; Mansourian et al., 2025a) that embed spheres of power, authority, strategic and technical capacity (Mermet et al., 2005; Peterson, Peterson & Peterson, 2005). The role of the different actors within the space of ecological restoration in Mauritius and Rodrigues is therefore defined by their capacity to act, embedding technical, scientific and operational capitals (Nicotra et al., 2015), and their legitimacy, whether claimed or imposed, to act (Meinard, 2017; Mansourian et al., 2025a), especially on controversial issues (Zachrisson & Lindahl, 2013).

Despite the fact that different approaches are being used, the landscape of restoration activities is still dominated by intensive over passive restoration techniques. Such a preference likely results from the islands having gone through rapid and extensive habitat destruction (Hammond et al., 2015) and severe impacts of introduced predators (Cheke & Hume, 2008) that led to high levels of species becoming threatened with extinction (Griffiths & Florens, 2006; Baider et al., 2010). This situation in turn encouraged intensive conservation projects that were successful in saving a few species from extinction, notably endemic birds (Jones, 2004; Jones & Merton, 2012). These successful projects, historically led by international organizations, have played a central role in legitimizing the implementation of intensive restoration activities in Mauritius. This history also helps explain the continued presence of key international organisations (Sawrey, Copsey & Milner-Gulland, 2019), alongside the enduring social, political and historical legacies of colonial and post-colonial Mauritius (Grove, 1996; Iranah et al., 2018).

Intensive conservation approaches, however, demand substantial resources. Over time, the growing presence and diversification of international donors at national and regional scales have gradually increased the amount of resources available in Mauritius and Rodrigues (AFD, 2025; CEPF, 2025; European Commission, 2025). This reshaped the islands conservation landscape by increasing the number of organisations involved, the range of actions undertaken, and the monitoring and evaluation frameworks. Nevertheless, we showed that financial constraints and staff capacity are still frequently mentioned as major obstacles to achieving practitioners’ restoration objectives. Past conservation successes through intensive and costly interventions, may have helped entrench a view that successful conservation requires intensive management, often species-centred. Although this may be true for some species (Asmussen-Lange, Maunder & Fay, 2011; Sarasan et al., 2015), it is evidently not for most species, which shifts from decline to recovery once threats (like posed by invasive introduced plants) are controlled, without additional human intervention (Florens et al., 2010; Baider & Florens, 2011; Probst & Florens, 2018; Probst, Florens & Ineich, 2018). Given that funding mechanisms and institutional arrangements strongly influence restoration activities, monitoring, and impact assessment (Enrici et al., 2023; Adamo et al., 2022; Craigie et al., 2015), it is crucial to understand why intensive and costly activities are still preferred over lower-cost alternatives that emphasise natural regeneration. Ultimately, this would help identifying ways to shift prevailing mindsets away from favouring overly intensive and expensive management (Crouzeilles et al., 2016; Chazdon et al., 2024).

In addition, although ecological monitoring represents one of the most implemented activities among practitioners, the assessment of ecological restoration success is hindered by a lack of standardisation in monitoring within restoration projects and a lack of in-built research that can support improvements. Indeed, only the survival and growth rate of native plant species (that are often planted) are reported as being monitored by most practitioners. The other parameters mentioned during interviews, such as native plant and animal recruitment, are monitored sporadically. Less than one third of practitioners mentioned monitoring potential socio-economic impacts of restoration activities (such as number of people employed under a project, number of people visiting restoration sites, or community feedback) but rarely do so systematically. Finally, a minority of practitioners (18.5%) considered research as a restoration activity. However, when research is not viewed as a restoration activity the understanding of success and the ability to properly adapt management is lessened. Restoration projects should therefore integrate meaningful monitoring and research (without which adaptive management remains weak or impossible), and be better aligned with national and international restoration targets for biodiversity conservation (Van Diggelen, Grootjans & Harris, 2001; Halle, 2007; Dickens & Suding, 2013; Mahajan et al., 2023).

In Mauritius and Rodrigues, the tendency to consider social and ecological impacts of restoration projects separately is another factor that can ultimately impact the type of restoration activities implemented, leading to management actions prioritising one element over the other. This situation can be attributed to a prevailing emphasis on land-sparing conservation strategies (Fischer et al., 2014), as has historically been the case on these islands. Such conservation strategies, characterised by predominantly exclusionary and preventive approaches and laws to protect terrestrial native biodiversity (Grove, 1996; Cheke & Hume, 2008), might however bias conservation practitioners’ decisions when balancing social and ecological considerations (Löfroth et al., 2024; Neeson, Emmons & Mullenbach, 2024), privileging certain values and worldviews (Agrawal & Redford, 2009; Brockington & Igoe, 2006; Cernea & Schmidt-Soltau, 2006; Martin, McGuire & Sullivan, 2013; West, Igoe & Brockington, 2006). Ultimately, such approaches risk reducing the ability to objectively evaluate whether chosen conservation methods are appropriate for meeting the intended objectives, thereby limiting or even precluding opportunities for improvement.

Finally, our study highlights the paradoxical difference between where challenges facing conservation practitioners arises, and where solutions that they foresee to improve success, applies. We showed that organisational and national issues are perceived by practitioners as a major challenge. Although practitioners reported implementing responsive activities such as fostering collaborations, enhancing organisation efficiency and staff welfare (under question 33b, see Supplemental Information 11), these activities were not reported as key solutions to improve restoration success. Instead, technical and methodological solutions were most frequently perceived as those improving restoration success (*N* = 135; 68%), compared to other types of solution (*N* = 64; 32%). By doing so, however, conservation practitioners might to some extent mitigate the major threats affecting biodiversity at the scale of their restoration sites, but miss opportunities to share knowledge and expertise, limit structuring restoration efforts at the more effective landscape scale, and fail to develop coordinated solutions for other predominant challenges faced at the national level. In such situations, conditions are not set for up-scaling restoration efforts which in turn curtail the chances to reach set national and international objectives. In Mauritius, this helps explain the divergence between the restoration target set in the 2017-2025 National Biodiversity Strategy and Action Plan (NBSAP) (Republic of Mauritius, 2017), and what has been achieved (Republic of Mauritius, 2024).

### Insights to improve restoration efforts in Mauritius and Rodrigues

Defining if implemented ecological restoration activities are effective (which is understood as how successful they are in meeting set objectives) ultimately depends on which success indicators are being measured (Bakker et al., 2000; Ruiz-Jaen & Aide, 2005; Gann et al., 2019; Holl, 2020). For Mauritius and Rodrigues, the National Biodiversity Strategy and Action Plan (NBSAP) 2017–2025 (Republic of Mauritius, 2017) serves as the national-level framework guiding ecological restoration efforts. However, although it defines many priority actions and targets, key performance indicators are lacking, and monitoring responsibilities are fragmented (Government of Mauritius, 2021). As a result, it becomes difficult to assess whether conservation measures are effective or being properly implemented. In addition, this framework does not provide the necessary mechanisms for stakeholders to exchange data, lessons learned, or coordinate actions, limiting long-term collaboration and institutional memory (Government of Mauritius, 2021). Such lack of coordination and collaboration between organisations has however barely been recognised as an issue in our study, accounting for less than 4% of reported challenges, although interpersonal issues, ego and jealousy issues, lack of trust between practitioners, and resource constraints are directly linked to it. Hence, enhancing the current national framework and raising awareness about its importance appears critical if the success of restoration on both islands is to be assessed thoroughly and national and international objectives are to be met.

Allowing naturally regenerating fast-growing native pioneer species to speed up ecological restoration following weeding activities has been repeatedly suggested as a better, faster, and cheaper restoration and conservation approach to restore native terrestrial habitats in Mauritius for more than one decade (Florens, 2013; Baider & Florens, 2022; Baguette, Baider & Florens, 2025a). Indeed, studies conducted on the island and regionally showed that it is better for native biodiversity, as it suppresses reinvasion by introduced plants (Florens & Baider, 2013; Otuoma et al., 2020) and fosters faster recovery of native epiphytes and lianas (Baguette, Baider & Florens, 2025b) among other benefits. In addition, it accelerates ecological restoration by rapidly closing the substantial canopy gaps created by the removal of invasive alien plants (Baider & Florens, 2011; Baguette, Baider & Florens, 2025a). Furthermore, this approach is cheaper as it reduces the need for intensive site management activities (Bellefontaine & Malagnoux, 2009; Martins, 2017). Although these benefits have been recognised by some conservation practitioners under this study (Supplemental Information 12), the use of pioneer trees for restoration remains undervalued in Mauritius and Rodrigues, as the use of pioneer trees is only seen as a possible solution by a minority of practitioners (7.4% of respondents). In contrast, some practitioners even actively remove pioneer trees from restoration sites to plant seedlings of slower growing native species instead (Florens & Baider, 2013), a number of which have a stem diameter growth rate of less than one mm per year (Baider & Florens, 2006; Strahm, 1993). It highlights that the use of assisted natural regeneration methods for restoration still begs substantial consideration, as is the case in other sectors like forestry or land management (Shono, Cadaweng & Durst, 2007; Brancalion et al., 2019; Chazdon et al., 2020). Resolving such ambiguities is, therefore, vital for the implementation of cost-effective restoration activities (Oluwajuwon et al., 2024), and is a crucial precondition to enable upscaling restoration on both islands.

Part of these contradictions might stem from the variety of organisations involved, which work in very different contexts, have different objectives, and hold different values. Hence, they often tend to work in silos, impeding the development of synergies and ultimately the improvement of restoration projects. Solutions perceived to improve the organisation’s restoration success are therefore mostly technical and focused on improving core activities being done locally, like weeding, planting, or plant propagation, but rarely address broader challenges that would improve restoration success nationally. However, such diversity of organisations involved in the ecological restoration of native terrestrial habitats could be organised into a strength for the islands if they were to collaborate. Indeed, different practitioners play different roles, and have access to different levels of resources available, including technical, that allow them to respond to challenges in different ways and based on their intrinsic project characteristics (Mohr & Metcalf, 2018; Chazdon et al., 2021; Schweizer, Van Kuijk & Ghazoul, 2021; Van Oosten, Runhaar & Arts, 2021; Beeton, Cheng & Colavito, 2022). Overcoming perceived barriers and challenges to collaboration will certainly bring more useful synergies, and ultimately improve the island’s ability to reach their national and international restoration objectives (Burt et al., 2022).

### Study strengths and limitations

For achievability, we restricted our survey to Mauritius and Rodrigues, two oceanic islands which have sustained extensive natural habitats degradation by human activities. Subsequent to this degradation, the islands have deployed biodiversity conservation and ecological restoration activities over the last decades, some of which resulted in globally acclaimed conservation successes. Mauritius and Rodrigues are models to study native habitats undergoing ecological restoration because they face threats to their biodiversity that are broadly shared by most other oceanic islands (Caujapé-Castells et al., 2010). Prevailing conditions and dynamics, particularly on Mauritius, can arguably be considered as conferring to it a status of microcosm of the world (Koenig et al., 2026). Despite such similarities, substantial variation still exists among islands, modulated for example by idiosyncratic functional shifts driven by introductions and extinctions (Heinen et al., 2025) or by whether human colonisation occurred during or before colonialism (Seetah et al., 2022). These differences would introduce limitations to our study because they would shape contrasting perceptions among practitioners, which are hard to circumvent anyway, but must be kept in mind. Consequently, ecological restoration activities implemented on islands would vary. Nevertheless, this study’s strength lies in its completeness, including perceptions and observational data from conservation practitioners working in contrasting sectors (public, private, NGO, and community members). This cross-sectional perspective, which remains scarce in the ecological restoration literature, provides a practice-oriented understanding of restoration challenges and opportunities. By capturing practitioner-driven insights grounded in the long-term, this study offers insights that can help support improvements of restoration strategies in comparable island contexts and contribute to addressing conservation challenges across multiple scales.

## Conclusion

We characterised the diversity of activities implemented to restore native terrestrial habitats in two oceanic islands reputed for several world acclaimed conservation successes and found an extensive diversity of perceived challenges faced by conservation practitioners. Active restoration activities, often involving expensive facilities, equipment, and significant human resources, dominate the ecological restoration landscape, while assisted natural regeneration methods, monitoring and research are neglected. Despite the extensive investments in ecological restoration projects, there exists little monitoring to assess the pertinence of the chosen ecological restoration approaches and where monitoring does occur, a lack of standardisation that limits its usefulness, as practitioners monitor mostly the growth and survival of (often planted) seedlings as ecological indicators and rarely including socio-economic parameters in their monitoring. In addition, a mismatch between perceived challenges hindering restoration practices and solutions to improve restoration success likely impedes improvement of restoration. Taken together, these limitations make the evaluation of ecological restoration success difficult and inherently subjective. Overall, our study allowed to highlight the relevance of small oceanic islands to study ecological restoration, and raises the need and importance to (1) clarify the vision and objectives of practitioners to assess how well they articulate and align with national and international objectives, and (2) shift the current ecological restoration approaches towards more cost-effective solutions that will enable the upscaling of ecological restoration that is required if sustainable conservation is to be achieved.

## Supplemental Information

10.7717/peerj.21687/supp-1 Supplemental Information 1 STROBE Checklist completed for this study

10.7717/peerj.21687/supp-2 Supplemental Information 2 English version of the questionnaire used for this study

10.7717/peerj.21687/supp-3 Supplemental Information 3 List of variables included in the final dataset of this study to describe the diversity of restoration activities implemented

10.7717/peerj.21687/supp-4 Supplemental Information 4 Coded dataset showing the 17 variables extracted to categorize the diversity of restoration practices implemented

10.7717/peerj.21687/supp-5 Supplemental Information 5 Coded dataset showing the list of perceived challenges faced by conservation practitioners with the level at which they arise and their respective number of mentions

10.7717/peerj.21687/supp-6 Supplemental Information 6 Coded dataset showing the list of solutions perceived to increase ecological success by conservation practitioners, with the level at which they apply and their respective number of mentions

10.7717/peerj.21687/supp-7 Supplemental Information 7 List of ecosystem types reported under restoration in Mauritius and Rodrigues in this study

10.7717/peerj.21687/supp-8 Supplemental Information 8 List of variables significantly contributing to the clustering of the ecological restoration practices identified (Fisher’s exact test *p* < 0.05)

10.7717/peerj.21687/supp-9 Supplemental Information 9 Coded observation data reported by 54 responding conservation practitioners in Mauritius and Rodrigues with their associated ecological restoration cluster number

10.7717/peerj.21687/supp-10 Supplemental Information 10 List of activities reported under this study to address dominant challenges hindering the implementation of restoration activities in Mauritius and Rodrigues

10.7717/peerj.21687/supp-11 Supplemental Information 11 Quotes extracted from interviewee’s responses under this study highlighting the cost-effectiveness of using pioneer trees within ecological restoration projects in Mauritius and Rodrigues

## Additional Information and Declarations

### Data Availability

The following information was supplied regarding data availability: The data is available in the Supplemental Files.
