# Supplementary material for: Practitioners’ perceptions on ecological restoration: activities, challenges and solutions on two small oceanic islands
Source: PeerJ. 2026 Sep 21;14:e21687. doi: 10.7717/peerj.21687 (PMC13603903; doi:10.7717/peerj.21687)
Supplement: Supplementary file 1 [file peerj-14-21687-s002.docx]

**QUESTIONNAIRE-BASED SURVEY**

**Assessment of the factors and considerations influencing ecological restoration practices of native terrestrial habitats in a small oceanic island.**

**Context:** This study (ethical clearance reference UoMREC/2024/P76) is being conducted by François Baguette, affiliated with the University of Mauritius as part of his MPhil/PhD in Tropical Ecology and Conservation. The questionnaire-based survey is focused on ecological restoration practices implemented in native terrestrial habitats in Mauritius. It seeks to understand the factors and considerations influencing ecological restoration on the island.

**All your answers are strictly confidential and will only be used for scientific purpose. Your identity and responses will not be disclosed to any authorities or organisations. Only the general and compiled results will be shared outside of the research group and these cannot be used to identify respondents.**

**Date: Stakeholder Category:**

**Time: Reference:**

**Part 1 – Respondent’s organisation profile**

*This section is focusing on your professional profile, your background with terrestrial ecological restoration and your current activities in this field.*

1. **What is the type of organisation that best describe the one you represent?**
2. **What is(are) your role(s) regarding ecological restoration?**

***Please select one or multiple option(s).***

- Funder
- Scientific advisor/ Researcher
- Restoration project implementation
- Policy regulation
- Other: ……………………………………
- I prefer not to answer

3. **What is the job title that best describe your role over the last three years of your career?**
4. **What is the highest education level you obtained?**
5. **What is the field of study of your highest education level?**
6. **During how many years have you been involved in ecological restoration and/or biodiversity conservation projects?**
7. **During how many years have your organisation/department been involved in ecological restoration and/or biodiversity conservation projects?**
8. **Which of the following categories best fit the size of your organisation/department in terms of number of staffs?**

- 1
- 2-10
- 10-20
- 20-30
- >30
- I prefer not to answer

9. **How many people are employed for restoration/conservation efforts within your organisation/department?**
a. Number employed full time
b. Number of seasonal workers (including volunteers and interns) per year?
10. **What is your/your organisation/department approximate annual budget for restoration/conservation efforts?**
11. **What is(are) your/your organisation/department main source(s) of funds for restoration?**

**Part 2 – Values and perception of native ecosystems**

*This section is focusing on your values and perceptions about nature and the native ecosystems of Mauritius.*

12. **As forests constitute a typical example of native ecosystems from Mauritius, what do Mauritian forests mean to you?**

***List all items which come to your mind [in the order they are given]. Use left-to-right separated by a comma to maximize use of space.***

13. **What values of nature are most important to you?**

***Please rank the values below from 1 to 3 (or 1 to 4 if you add an additional value, 1 being the most important).***

| Value | Rank |
| --- | --- |
| a. The goods and the services it provides to humans, including food, medicine, income, etc… |  |
| b. The relationship I have created with natural spaces and organisms. |  |
| c. Its intrinsic and stand-alone value, it does not have to be related to humans to be important. |  |
| d. Other: …………………… |  |

14. **What would your ideal forest look like?**

***Please give five of its key features.***

**Part 3 – Restoration practices**

*This section is designed to assess ecological restoration practices implemented in Mauritius*

15. **What does ecological restoration mean to you?**
16. **Read the following statements and decide to what extent you agree with each.**

*1: Disagree	2: Neither agree nor disagree	3: Agree	0: I prefer not to answer*

| a. Ecological restoration aims to save the ecosystem or site, not necessarily in its current state, but as a dynamic system. | 1 | 2 | 3 | 0 |
| --- | --- | --- | --- | --- |
| b. Ecological restoration aims at making certain changes to the current state of the managed ecosystem or site with the goal of bringing it back to earlier conditions. | 1 | 2 | 3 | 0 |
| c. Ecological restoration involves the active and intentional human modification of a managed area, so that ecosystems are not necessarily engineered to be similar to ecosystems or sites that existed before. | 1 | 2 | 3 | 0 |
| d. One should not interfere with nature at all, even in ecological restoration. | 1 | 2 | 3 | 0 |
| e. One has no responsibility to assist nature but it is permissible to assist if needed. | 1 | 2 | 3 | 0 |
| f. It is our duty to intervene with nature. | 1 | 2 | 3 | 0 |

17. **What are the objectives you seek to achieve through restoration efforts?**

***Please rank the categories below from 1 to 5 (or 1 to 6 if you add an additional category, 1 being the most important).***

| Categories of objectives | Rank |
| --- | --- |
| a. Biodiversity and ecosystem conservation |  |
| b. Climate change mitigation/compensation |  |
| c. Landscape/aesthetic value |  |
| d. Livelihoods’ improvement, social benefits provision, and community empowerment |  |
| e. Production of timber, food, craft or medicine |  |
| f. Other: …………………………………… |  |

18. **What process do you follow to define your restoration objectives? Please elaborate.**
19. **What source of information do you primarily use to define your objectives?**
20. **What are the restoration activities that you implement? Please elaborate with details.**
21. **Do you monitor any ecological parameter to assess the success of your restoration activities?**

- Yes
- No
- I don’t know

22. **If so, could you cite three parameters you use to assess ecological restoration success?**
23. **Do you monitor the socio-economic impacts of the restoration activities you are involved with?**

- Yes
- No
- I don’t know

24. **If so, could you cite three indicators you use to assess their socio-economic impacts?**
25. **Approximately how much area (in ha) do you or your organisation currently manage for conservation or restoration purposes?**
26. **(a) Are your restoration efforts implemented in areas under some type of designation (legal, leased, or part of an overarching denomination [e.g. UNESCO World Heritage Site, UNESCO Biosphere Reserve])?**

- Yes
- No

(b) **Could you please specify which one(s) if it is the case?**
27. **Which category(ies) best describe(s) the ecosystem(s) you are restoring/conserving?**

***Please select one or multiple option(s).***

- Coastal wetland (marsh, mangrove, natural pond)
- Coastal vegetation / islets (e.g. Ile aux Aigrettes, Ilot Gabriel)
- Mainland lowland vegetation (0-200 m a.s.l., )
- Intermediate vegetation (200-400 m a.s.l)
- Upland wetlands (>400 m a.s.l.)
- Upland forest
- Upland heath

28. **What are the main threats impacting the ecosystem(s) you are involved with? Rate their severity between mild, moderate and severe.**

| **Threat** | **Mild** | **Moderate** | **Severe** |
| --- | --- | --- | --- |
|  |  |  |  |
|  |  |  |  |
|  |  |  |  |
|  |  |  |  |
|  |  |  |  |
|  |  |  |  |
|  |  |  |  |
|  |  |  |  |

29. **Which of these threats mobilize most of your financial and human resources?**

**Part 4 – Challenges and constraint for restoration success**

*This section is designed to assess factors influencing ecological restoration of native terrestrial habitats in Mauritius*.

30. **How would you evaluate the success of your restoration efforts?**

- No success
- Low
- Medium
- High
- Other (please specify): …………………………………….

31. **How do you define ecological restoration success? Please elaborate**
32. **Can you cite and rank the five most important challenges you face to reach your restoration effort objectives?**
33. **(a) Do you implement responsive actions to answer to the challenges you face to reach your restoration objectives?**

- Yes
- No

**(b) Can you specify which ones if it is the case?**

34. **How would you rate your current level of collaboration with other organisations in Mauritius?**

- No collaboration
- Low
- Medium
- High
- Other (please specify): ………………………………..

35. **(a) What challenges have you encountered to develop in-country collaboration on restoration in Mauritius?**

**(b) Please elaborate on the reasons why you faced those challenges.**

36. **What opportunities have you gained from the in-country collaboration you developed? Please elaborate**
37. **How would you rate your current level of collaboration with other organisations outside Mauritius?**

- No collaboration
- Low
- Medium
- High
- Other (please specify): ………………………………..

38. **(a) What challenges have you encountered to develop international collaboration?**

**(b) Please elaborate on the reasons why you faced those challenges.**

39. **What opportunities have you gained from the international collaborations you developed? Please elaborate**
40. **(a) Has the legislative and policy landscape in Mauritius been a driver to improve your restoration success?**

- Yes
- No
- I don’t know

**(b) Please explain why in the box below**

41. **(a) Have the funding opportunities available for restoration in Mauritius been a driver of your restoration success?**

- Yes
- No
- I don’t know

**(b) Please explain why in the box below**

42. **How would you evaluate your current technical capacity to reach restoration success?**

- No internal capacity
- Low
- Medium
- High
- Other (please specify): …………………………………….

43. **(a) What challenges have you encountered to reach sufficient technical capacity for restoration success in Mauritius?**

**(b) Please explain why in the box below**

44. **How would you evaluate your current organisation on-field staff capacity to reach restoration success?**

- No on field capacity
- Low
- Medium
- High
- Other (please specify): ………………………………..

45. **(a) What challenges have you encountered to reach sufficient on-field capacity for restoration success in Mauritius?**

**(b) Please explain why in the box below**

**Part 5 – Avenues to improve ecological restoration success in Mauritius**

*This section is designed to assess solutions that could improve ecological restoration success in Mauritius*

46. **(a) Have you identified any practices and/or tools that improved your restoration success?**

***Please specify which ones and how they improve your restoration success.***

(b) **Have you identified challenges to integrate these practices and/or tools into your restoration efforts? Please elaborate.**
47. **In the table below, please list any other tools or practices that would improve your restoration success but you could not implement and the constraints you are facing?**

| **Restoration practice / tool** | **Main constraints for implementation** |
| --- | --- |
|  |  |
|  |  |
|  |  |
|  |  |
|  |  |
|  |  |
|  |  |

**Part 6 – Use of plant species as an ecological restoration tool in Mauritius**

*This section is designed to assess solutions, specifically the use of pionner species as a tool that could improve ecological restoration success in Mauritius*

48. **Do you know what a pioneer plant species is?**

- Yes
- No

49. **How would you define a pioneer plant species?**
50. **What are the main characteristics of pioneer plant species?**
51. **Could you cite up to five pioneer plant species present in Mauritius?**

| **Common name** | **Scientific name (if know)** | **Habitat** | **Status**  **(endemic-native/exotic- alien )** |
| --- | --- | --- | --- |
|  |  |  |  |
|  |  |  |  |
|  |  |  |  |
|  |  |  |  |
|  |  |  |  |

52. **(a) Do you believe that integrating native pioneer plant species use within your restoration efforts could improve their success?**

- Yes
- No
- I don’t know
- Other: ……………………………………………………

(b) **If yes, please specify why.**
53. **(a) Do you currently have a management strategy regarding pioneer plant species?**

- Yes
- No
- I don’t know

**(b) If so, could you please explain what it consists of?**

54. **(a) Do you have costs associated to native pioneer plant species management in your restoration efforts?**

- Yes
- No
- I don’t know

**(b) If yes, could you please elaborate?**

**(c) Could you please provide an estimate of these costs?**

55. **Do you know about *Harungana madagascariensis*?**

- Yes
- No

56. **(a) Is *Harungana madagascariensis* present within the site you work in?**

- Yes
- No
- I don’t know

**(b) If yes, how do you manage this species? Please elaborate.**

57. **Do you have any additional comment(s) on *Harungana madagascariensis*?**
58. **Do you have any additional other comment(s) you would like to add?**

*Thank you very much for participating to this survey.*

*If you have questions, please use the contact information in the consent form.*

*If you want to be informed on our findings from this study, please use the contact information in the consent form to reach out to us.*
