# Supplementary material for: Practitioners’ perceptions on ecological restoration: activities, challenges and solutions on two small oceanic islands
Source: PeerJ. 2026 Sep 21;14:e21687. doi: 10.7717/peerj.21687 (PMC13603903; doi:10.7717/peerj.21687)
Supplement: Supplementary file 2 [file peerj-14-21687-s008.docx]

| Variable | df | p_value |
| --- | --- | --- |
| Planting | 3 | **1.12E-11** |
| Plant species selection | 6 | **7.85E-10** |
| Predator control | 3 | **2.23E-9** |
| Density of planting | 12 | **1.16E-8** |
| Conduct fauna conservation programs | 3 | **3.43E-7** |
| Planting after-care | 3 | **4.72E-5** |
| Propagation of plant species | 3 | **7.93E-5** |
| Conduct research | 3 | **1.01E-4** |
| Weeding of invasive plants | 3 | **1.57E-4** |
| Use of fencing | 3 | **8.55E-4** |
| Using pioneer species as restoration strategy | 6 | **1.35E-3** |
| Ecological monitoring implementation | 6 | **1.15E-2** |
| Advice on restoration technique | 3 | **4.03E-3** |
| Policy development and law implementation | 3 | 5.37E-2 |
| Socio-economic monitoring implementation | 3 | 1.05E-1 |
| Soil preparation | 3 | 3.55E-1 |
| Conduct education campaigns and training | 3 | 6.86E-1 |
