# Supplementary material for: Practitioners’ perceptions on ecological restoration: activities, challenges and solutions on two small oceanic islands
Source: PeerJ. 2026 Sep 21;14:e21687. doi: 10.7717/peerj.21687 (PMC13603903; doi:10.7717/peerj.21687)
Supplement: Supplementary file 3 [file peerj-14-21687-s001.doc]

STROBE Statement—Checklist of items that should be included in reports of ***cross-sectional studies***

This checklist follows the Strengthening the Reporting of Observational Studies in Epidemiology (STROBE) guidelines. It summarizes how each recommendation has been addressed in the manuscript entitled “Practitioners’ perceptions on ecological restoration: Practices, challenges and solutions on two small oceanic islands.”

|  | Item No | Recommendation | Respected ? | Location in manuscript |
| --- | --- | --- | --- | --- |
| **Title and abstract** | 1 | (*a*) Indicate the study’s design with a commonly used term in the title or the abstract | Yes | Study design is indicated in the abstract line 30-34  “Using a questionnaire-based survey on two well-known oceanic islands in the fields of terrestrial ecological restoration and conservation (Mauritius and Rodrigues in the Mascarenes), we explored (1) the modalities of restoration practices of native terrestrial habitats for biodiversity conservation, (2) challenges impacting their implementation, and (3) potential solutions from conservation practitioners’ perspectives.” |
|  |  | (*b*) Provide in the abstract an informative and balanced summary of what was done and what was found | Yes | This information is stated in the study abstract (study objective described, method and results described) line 22-50 |
| Introduction | | |  |  |
| Background/rationale | 2 | Explain the scientific background and rationale for the investigation being reported | Yes | Rationale and existing literature are stated in the introduction section |
| Objectives | 3 | State specific objectives, including any prespecified hypotheses | Yes | A statement at the end of the introduction specifies the specific goals and objectives in line 126-131.  “In this study, we adopt a structured questionnaire-based survey with conservation practitioners from different sectors (government institutions, non-governmental organizations (NGOs), private sector, individuals, academia) to explore (1) the modalities of practices adopted to restore native terrestrial habitats, using the small oceanic islands of Mauritius and Rodrigues as case studies; (2) the challenges they face; and (3) the potential solutions that could be brought based on practitioner’s perceptions.” |
| Methods | | |  |  |
| Study design | 4 | Present key elements of study design early in the paper | Yes | Study design is stated in the data collection section of the Material and Methods section in line 163-194. |
| Setting | 5 | Describe the setting, locations, and relevant dates, including periods of recruitment, exposure, follow-up, and data collection | Yes | Setting, locations, and relevant dates are described in the data collection section of the Material and Methods section in line 163-194. |
| Participants | 6 | (*a*) Give the eligibility criteria, and the sources and methods of selection of participants | Yes | Eligibility criteria and the sources and methods of selection of participants are described in the data collection section of the Material and Methods section in line 163-194. |
| Variables | 7 | Clearly define all outcomes, exposures, predictors, potential confounders, and effect modifiers. Give diagnostic criteria, if applicable | Yes | Detailed explanation on raw data coding is available in the data analysis section of the Material and Methods section in line 195-241. |
| Data sources/ measurement | 8* | For each variable of interest, give sources of data and details of methods of assessment (measurement). Describe comparability of assessment methods if there is more than one group | Yes | Detailed explanation on coded data analysis is available in the data analysis section of the Material and Methods section in line 195-241. |
| Bias | 9 | Describe any efforts to address potential sources of bias | Yes | We notably tried to reduce bias by excluding the least mentioned restoration practices variables to reduce noise in the data as much as possible. Such procedure is explained in the Material and Methods section in line 205-208. |
| Study size | 10 | Explain how the study size was arrived at | Yes | The total number of interviewees responses included in the final analysis is included in the first paragraph of the Result section in line 243-252. |
| Quantitative variables | 11 | Explain how quantitative variables were handled in the analyses. If applicable, describe which groupings were chosen and why | Yes | The way all variables were handled in the analysis is explained in the data analysis section of the Material and Methods section in line 195-241. |
| Statistical methods | 12 | (*a*) Describe all statistical methods, including those used to control for confounding | Yes | All statistical methods used in this study are explained in the data analysis section of the Material and Methods section in line 195-241. |
|  |  | (*b*) Describe any methods used to examine subgroups and interactions | Yes | The method used in this study to examine clusters of restoration practices is explained in the data analysis section of the Material and Methods section in line 195-241. |
|  |  | (*c*) Explain how missing data were addressed | Yes | All participants of this study responded to all questions of the questionnaire, hence there is no actual missing data. If participants could not answer certain questions, these answers were recorded as “unknown”. Coded data available in supplementary material present all data recorded from all participants. |
|  |  | (*d*) If applicable, describe analytical methods taking account of sampling strategy | NA | Non applicable |
|  |  | (*e*) Describe any sensitivity analyses | NA | Non applicable |
| Results | | |  |  |
| Participants | 13* | (a) Report numbers of individuals at each stage of study—eg numbers potentially eligible, examined for eligibility, confirmed eligible, included in the study, completing follow-up, and analysed | Yes | This is described at the beginning of the result section in line 243-252. |
|  |  | (b) Give reasons for non-participation at each stage | Yes | This is described at the beginning of the result section in line 243-252. |
|  |  | (c) Consider use of a flow diagram | NA | Use of a flow diagram was not deemed appropriate |
| Descriptive data | 14* | (a) Give characteristics of study participants (eg demographic, clinical, social) and information on exposures and potential confounders | Yes | Study participants characteristics are presented in the first paragraph of the result section in line 243-252. |
|  |  | (b) Indicate number of participants with missing data for each variable of interest | Yes | All participants of this study responded to all questions of the questionnaire, hence there is no actual missing data. Coded data present all data recorded from all participants. |
| Outcome data | 15* | Report numbers of outcome events or summary measures | Yes | All numbers are reported in Tables |
| Main results | 16 | (*a*) Give unadjusted estimates and, if applicable, confounder-adjusted estimates and their precision (eg, 95% confidence interval). Make clear which confounders were adjusted for and why they were included | NA | Non-adjusted frequencies were not displayed in the interest of table clarity. However, the effect of adjustment on frequency did not impact on final data analysis results. |
|  |  | (*b*) Report category boundaries when continuous variables were categorized | NA | Non applicable |
|  |  | (*c*) If relevant, consider translating estimates of relative risk into absolute risk for a meaningful time period | NA | Non applicable |
| Other analyses | 17 | Report other analyses done—eg analyses of subgroups and interactions, and sensitivity analyses | NA | Non applicable |
| Discussion | | |  |  |
| Key results | 18 | Summarise key results with reference to study objectives | Yes | Key results and their implications for biodiversity conservation and ecological restoration are discussed throughout the discussion section. |
| Limitations | 19 | Discuss limitations of the study, taking into account sources of potential bias or imprecision. Discuss both direction and magnitude of any potential bias | Yes | Description of limitations is done under “Strengths and limitations” heading in the discussion in line 452-473. |
| Interpretation | 20 | Give a cautious overall interpretation of results considering objectives, limitations, multiplicity of analyses, results from similar studies, and other relevant evidence | Yes | References were added where possible, and discussed. Limitations were taken into account in the discussion in line 452-473. |
| Generalisability | 21 | Discuss the generalisability (external validity) of the study results | Yes | Study results were deemed generalizable at Global level, and discussed as such. |
| Other information | | |  |  |
| Funding | 22 | Give the source of funding and the role of the funders for the present study and, if applicable, for the original study on which the present article is based | Yes | Funding information were displayed upon submission but not included in the manuscript, as requested. |

*Give information separately for exposed and unexposed groups.
